# Iron bioleaching and polymers accumulation by an extreme acidophilic bacterium

**DOI:** 10.1007/s00203-024-04005-4

**Published:** 2024-05-22

**Authors:** Alessandro Marchetti, Daniel Kupka, Vittorio Giorgio Senatore, Zuzana Bártová, Paola Branduardi, Lenka Hagarová, Slavomír Hredzák, Marina Lotti

**Affiliations:** 1 Department of Biotechnology and Biosciences, State University of Milano-Bicocca, Milano, Italy; 2 Institute of Geotechnics of the Slovak Academy of Sciences, Watsonova 45, Kosice, 040 01 Slovakia

**Keywords:** Bioleaching, Acidophiles, *Acidiphilium*, Poly-β-hydroxybutyrate (PHB), Extremophiles

## Abstract

In many European regions, both local metallic and non-metallic raw materials are poorly exploited due to their low quality and the lack of technologies to increase their economic value. In this context, the development of low cost and eco-friendly approaches, such as bioleaching of metal impurities, is crucial. The acidophilic strain *Acidiphilium* sp. SJH reduces Fe(III) to Fe(II) by coupling the oxidation of an organic substrate to the reduction of Fe(III) and can therefore be applied in the bioleaching of iron impurities from non-metallic raw materials. In this work, the physiology of *Acidiphilium* sp. SJH and the reduction of iron impurities from quartz sand and its derivatives have been studied during growth on media supplemented with various carbon sources and under different oxygenation conditions, highlighting that cell physiology and iron reduction are tightly coupled. Although the organism is known to be aerobic, maximum bioleaching performance was obtained by cultures cultivated until the exponential phase of growth under oxygen limitation. Among carbon sources, glucose has been shown to support faster biomass growth, while galactose allowed highest bioleaching. Moreover, *Acidiphilium* sp. SJH cells can synthesise and accumulate Poly-β-hydroxybutyrate (PHB) during the process, a polymer with relevant application in biotechnology. In summary, this work gives an insight into the physiology of *Acidiphilium* sp. SJH, able to use different carbon sources and to synthesise a technologically relevant polymer (PHB), while removing metals from sand without the need to introduce modifications in the process set up.

**Supplementary Information:**

The online version contains supplementary material available at 10.1007/s00203-024-04005-4.

## Introduction

Extremophilic organisms are defined by their ability to thrive in harsh environmental conditions, regarding temperature, salinity, pressure, and pH, among others. These organisms populate hostile habitats and are endowed with ad hoc physiological and molecular features that allow adaptation to the specific environment (Rothschild and Mancinelli 2001).

Acidophiles can survive at the very low pH values found in natural environments or generated by human activities, such as mining. Indeed, many acidophilic bacteria have been isolated from mine drainage waters where pH values can approach 2 (Baker-Austin and Dopson 2007). In this context, some extreme acidophilic bacteria are described as capable of performing the dissimilatory reduction of ferric ion (from Fe^3+^ to Fe^2+^) which is relevant for bioleaching, that is the exploitation of the metabolic activities or metabolic products of specific microorganisms for the extraction of metals. Quartz sand (or silica sand), derived from the erosion of quartz rock, is largely used as a non-metallic raw material in industry, from low value applications such as glass manufacturing, foundry casting, ceramic, and adhesive filler to high-added value applications such as silicon-metal wafers, optical glasses and photovoltaic panels (Sajjad et al. 2019; Brierley 2010; Platias et al. 2014). A major problem in the use of quartz sand is the presence of sulphides and iron-oxide compounds, such as goethite, hematite, or siderite, which are considered impurities and indeed compromise the qualities and properties of the sand itself (Platias et al. 2014). This has fostered the development of technologies to reduce or eliminate the presence of ferrous compounds in the raw material (Zhang et al. 2012; Du et al. 2011; Panda et al. 2015a). With the aim to improve the technological properties of materials, physical (electromagnetic and gravitational separation) and chemical (leaching with inorganic and organic acids and reductants) methods of mineral processing have been applied. Since chemical-physical methods, e.g. hydrometallurgy are expensive and energy demanding, as well as seriously polluting water, air and soils (Veglio’ 1997; Brierley 2010; Asghari et al. 2013; Panda et al. a, b), bioleaching has become increasingly popular over the years as an economical, eco-friendly and sustainable method for leaching of impurities from raw materials (Bosecker 1997; D’Hugues et al. 2008; Anjum et al. 2010, 2012; Brierley 2010; Sajjad et al. 2019; Srichandan et al. 2019; Wang et al. 2020).

Candidate micro-organisms for bioleaching differ in metabolic requirements (chemolithotrophic or chemoorganotrophic), growth temperature (mesophiles or thermophiles), pH conditions (acidophiles or neutrophiles), and oxygen dependence (Clark and Norris 1996; Kelly and Wood 2000; Norris et al. 2000; Johnson and Bridge 2002; Ohmura et al. 2002; Rohwerder et al. 2003). In recent years, several genera of bacteria and archaea have been characterised as suitable for iron bioleaching purposes, including chemolithoautotrophic strains (i.e. *Acidiothiobacillus sp.*) and heterotrophic bacteria such as *Acidiphilium* (Harrison 1981; Malik and Hedrich 2022; Johnson and McGinness 1991a; Johnson and Bridge 2002). These acidophilic heterotrophic microorganisms are known to reduce Fe(III) to Fe(II) under different oxygenation conditions by coupling the oxidation of an organic substrate to the reduction of Fe(III) (Küsel et al. 1999; Li et al. 2020a; Bridge and Johnson 2000; Kupka et al. 2007; Vašková and Kupka 2007). *Acidiphilium* sp. SJH was isolated from an acidic mine in North Wales and shown to be able to reduce ferric ion under different conditions and to bioleach the iron fraction derived from quartz sand (Bridge and Johnson 2000; Kupka et al. 2007; Vašková and Kupka 2007; Johnson et al. 1979).

This work aims to address the metabolic abilities of this acidophilic bacterium, studying its growth kinetics and the reduction of ferric iron in quartz sand in the presence of different carbon sources and growth conditions. Moreover, Fourier-transform infrared spectroscopy analysis (FTIR) was performed on whole *Acidiphilium* sp. SJH cells to define if and how the process affects their cellular response and the overall macromolecular composition. This approach has been applied successfully to study changes induced by various stressors as for example the overproduction of recombinant proteins and protein aggregation (Doglia et al. 2008; DeDivitiis et al. 2023).

## Materials & methods

### Strains and materials

*Acidiphilium* sp. SJH (NCIMB 12,826) was kindly provided by Prof. David Barrie Johnson (Bangor University, UK) and maintained in autoclave-sterilised acidic liquid medium (pH 2.5) composed of tryptic soy broth 0.26 g/L, (NH_4_)_2_SO_4_ 12.5 g/L, MgSO_4_·7H_2_O 5 g/L, galactose 2 g/L, salt mix 1 mL for 1 L medium. The composition of the salt mix was as follows: Na_2_HPO_4_ 177 g/L, KH_2_PO_4_ 170 g/L, NH_4_Cl 133 g/L, Na_2_SO_4_ 35.5 g/L, ZnSO_4_·7 H_2_O 10 g/L, CuSO_4_·5 H_2_O 1 g/L, MnSO_4_·H_2_O 0.76 g/L, CoSO_4_·7 H_2_O 1 g/L.

The sample of aeolian quartz sand from Šajdíkove Humence deposit (west Slovakia), hereafter named “as received”, was kindly provided by KERKOSAND, Ltd together with information about grain size ranging from 0.3 to 1.4 mm (mean grain diameter d_50_ = 0.6 mm). Quartz sand was autoclave-sterilised before bioleaching experiments.

Furthermore, the heavy fraction of the raw material containing coloring oxides was separated by gravity using bromoform (CHBr_3_) and the light fraction was further treated by bacterial leaching. The light fraction still contained residual iron impurities in quantities that exceeded the limits for the input raw material for glass production. Chemical analyses of the quartz sand and the separated light fraction (Table 1) was done by Atomic Absorption Spectrometer (AAS Varian AA-30), after dissolving the solid sample in aqua regia using a microwave digestion system (Berghof).

**Table 1:** Chemical composition of quartz sand samples that were subjected to bleaching (w/w)

Sample	SiO_2_	Fe_2_O_3_	K_2_O	Na_2_O	CaO	MgO	Al_2_O_3_	ZnO	MnO	TiO_2_	Cr_2_O_3_
	%	%	%	%	%	%	%	%	%	%	%
“As received”	96.32	0.32	0.54	0.40	0.44	0.35	1.55	0.0019	0.0146	0.0387	0.0017
Light fraction	96.94	0.13	0.54	0.34	0.42	0.32	1.27	0.0017	0.0027	0.0308	0.0007

### Bacterial growth

*Acidiphilium* sp. SJH cultures, maintained in liquid broth, were inoculated at 0.1 OD_600_ in the above-mentioned medium supplemented with different carbon sources: galactose (2 g/L), glycerol (2 g/L) or glucose (2, 5–10 g/L) and incubated at 30 °C, shaking at 180 rpm, in fully aerobic conditions. Growth was monitored by measuring the optical density (OD_600_) for up to 14 days. Duplication time was estimated from OD_600_ measurements. First, µ_MAX_ was calculated as the slope of the regression line of ln (OD_600_) of samples in exponential phase; then t_D_ was obtained as ln(2)/µ_MAX_. All experiments were performed in triplicate.

Consumption of carbon sources was monitored over time for up to 14 days by High-Performance Liquid Chromatography (HPLC, Agilent 1100/1200, Agilent Technologies, Inc.). The instrument was equipped with a Rezex™ ROA-Organic Acid H+ (8%) 300 × 7.8 mm column (Phenomenex). The column was kept at 40 °C and 0.005 N H_2_SO_4_ was pumped isocratically at a flow rate of 0.5 mL/min. Signals were detected by a refractive index detector (RID) and peaks were identified by comparison with known standards. Before analysis, each sample was centrifuged at 13,000 g for 10 min, the pH was adjusted to neutrality with NaOH 0.2 M and filtered with a 0.22 μm syringe filter.

O_2_ consumption and CO_2_ production during bacterial growth were measured using on-line gas analysis. Cultures were grown in magnetically stirred and adequately aerated reaction vessels with a working volume of 0.5 L. Under fully aerobic conditions oxygen was continuously supplied by air sterilized by filtration through a 0.22 μm pore size filter passing through the reactor headspace above the liquid. On-line measurement of oxygen consumption and carbon dioxide production from the gas-phase was used to accurately measure oxidation of the organic substrate. For baseline concentration of zero CO_2_ and stable O_2_ (20.95% vol.), a source of compressed dry “zero air” was used. Air was directed to reactors through mass flow controllers (Bronkhorst) to maintain a constant standard temperature and pressure (STP)-corrected flow rate of the incoming air. To eliminate evaporative water loss from the cultures, the air entering the reactors was re-humidified using Nafion® tubing submerged in deionized water. The gas multiplexer (Sable Systems International) was used for automatic switching of the air flow between baseline air and headspace gas of individual reaction flasks. The exhausted gas was scrubbed again with water vapour by combined Nafion membrane/anhydrone drying before analysis. Paramagnetic oxygen analyser and infrared carbon dioxide analyser (Sable Systems) were used for O_2_ and CO_2_ analyses in inlet and outlet air respectively. The analysers were calibrated for zero (99.99% N_2_) and ambient air O_2_ and CO_2_ concentrations span. The bioreactor temperature was controlled by means of a circulating water bath thermostat.

### Bioleaching of iron impurities from quartz sand

Leaching of quartz sand was carried out at 40% w/v final concentration in 100 mL flasks, testing different carbon sources, aeration conditions (microaerobic, aerobic, and full aerobic condition) and phase of growth of the bacterial culture (either exponential or stationary). Aeration of the cell cultures, adjusted according to the work of Johnson et al. (Johnson and McGinness 1991a; Johnson and Bridge 2002), was modulated through two parameters: the ratio between the culture volume and the total volume of the flask and agitation. For microaerobic cultures, cells were inoculated into 100 mL flasks filled with 100 mL of medium and quartz sand and incubated at 25 °C without shaking. In aerobic condition flasks contained 50 ml of the mix of quartz sand and cells culture and were incubated at 25 °C under 180 rpm shaking. The full aerobic condition was obtained with 20 mL culture incubated at 25 °C and 180 rpm shaking.

Exponential phase bioleaching was performed with cells pre-cultured under aerobic conditions, up to the exponential phase (0.5 OD_600_) and then inoculated into fresh medium with the same composition supplemented with 40% (w/v) quartz sand at 0.2 OD_600_. Stationary phase bioleaching was performed by pre-growing the cells under aerobic conditions up to the stationary phase (1.8 OD_600_). Subsequently, quartz sand was added up to 40% w/v.

Samples of the leaching liquors were taken in regular intervals through the cap’s septum by using sterile needles and syringes. The samples were filtered through membrane filters and immediately stabilized with sulphuric acid to avoid Fe^2+^ oxidation. Ferric iron concentration in the leaching liquors was determined by a UV-spectrophotometric method at 300 nm (Basaran and Tuovinen 1986). For ferrous iron detection, several colorimetric methods have been described (Yue et al. 2016; Bas 2021). Here, ferrous iron concentrations were determined by the modified o-phenanthroline spectrophotometric method, insensitive to Fe(III) interference (Herrera et al. 2007).

### Whole- cell fourier-transform infrared spectroscopy (FTIR) analyses

*Acidiphilium* sp. SJH cells were cultured for two weeks under bioleaching conditions (microaerobic conditions, galactose medium, 40% quartz sand). Bacterial cell samples were collected at time 0 and after 1 and 2 weeks of growth, pelleted by centrifugation at 3,500 g for 5 min and washed in physiological solution (NaCl 0.9%). This washing step was repeated 3 times to remove any trace of medium components. Cells were then resuspended in an appropriate volume of physiological solution prior to FTIR measurements. Cell suspensions (3 µL) were deposited onto a BaF2 window, transparent to IR, and dried at room temperature for at least 30 min to eliminate the excess of water (Ami et al. 2022). FTIR absorption spectra were acquired in transmission mode, in the 4000–700 cm^− 1^ spectral range, by a Varian 610-IR infrared microscope coupled to the Varian 670-IR FTIR spectrometer (both from Varian Australia Pty Ltd., Mulgrave VIC, Australia) and equipped with a nitrogen-cooled mercury cadmium telluride detector (Ami et al. 2021, 2022). The variable microscope aperture was adjusted to ~ 200 μm × 200 μm (spatial resolution). Measurements were performed at 2.0 cm^− 1^ spectral resolution, 25 kHz scan speed, triangular apodization, and by the accumulation of 512 scan co-additions. Spectra were normalized to the Amide I band area and the second derivative analysis was performed (after a 13-point smoothing of the measured spectra) by the Savitzky–Golay method (3rd polynomial, 9 smoothing points), using the GRAMS/32 software (Galactic Ind. Corp., Salem, NH, USA). For each sample, several spectra were collected by selecting different areas on the same sample through the variable diaphragm aperture of the infrared microscope. The control condition was set up by cultivating *Acidiphilium* sp. SJH cells under bioleaching conditions but without quartz sand. To evaluate the reproducibility of the results, two independent experiments were performed.

## Results

### Microbial growth kinetics on different carbon sources

**Fig. 1:**
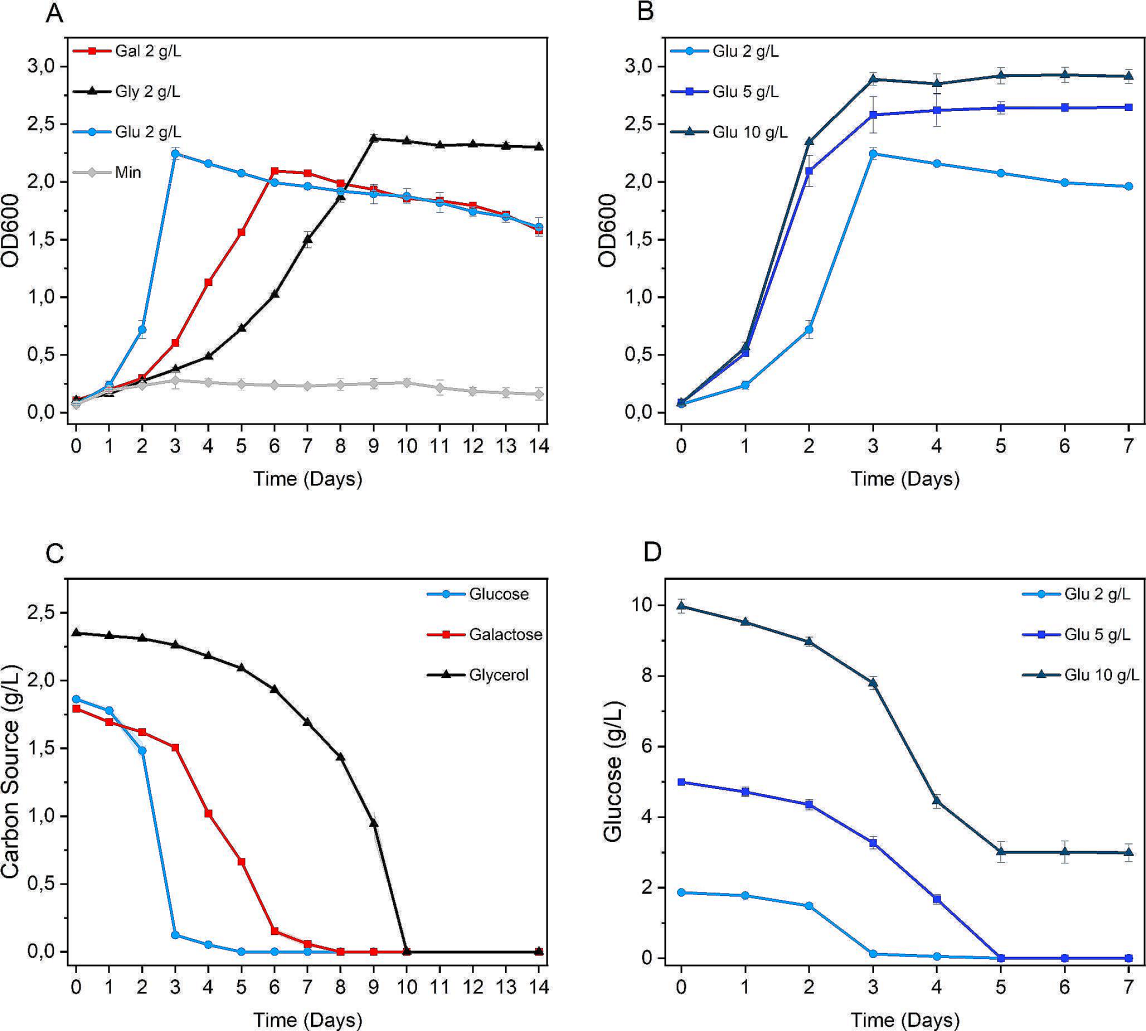
*Acidiphilium* sp. SJH growth and consumption of different carbon sources (**A** and **C**) and different glucose concentrations (**B** and **D**). Measurements were performed in triplicate and reported as a mean ± standard deviation

In medium containing glucose at 2 g/L, the culture showed a duplication time of 14.6 h and reached a maximum OD_600_ of 2.24 after 3 days (Fig. 1A). In galactose containing broth, the observed duplication time was 34.3 h and OD_600_ 2.09 was reached in 6 days. In the medium added with glycerol the duplication time was 48.3 h and the highest density of 2.37 OD_600_ was measured after 9 days cultivation. The faint growth observed in minimal medium without any added carbon source was probably supported by carry-over of residual nutrients from the pre-culture.

As glucose turned out to be the more effective carbon source for growth, it was tested at higher concentrations to boost biomass accumulation. As shown in Fig. 1B, in 5 g/L glucose the culture grew with a duplication time of 14.3 h and reached a maximum OD_600_ of 2.6. In medium containing 10 g/L glucose, cells displayed a duplication time of 13.7 h and reached 2.9 OD_600_. At all glucose concentrations tested, the culture entered the stationary phase after the third day of growth.

**Fig. 2:**
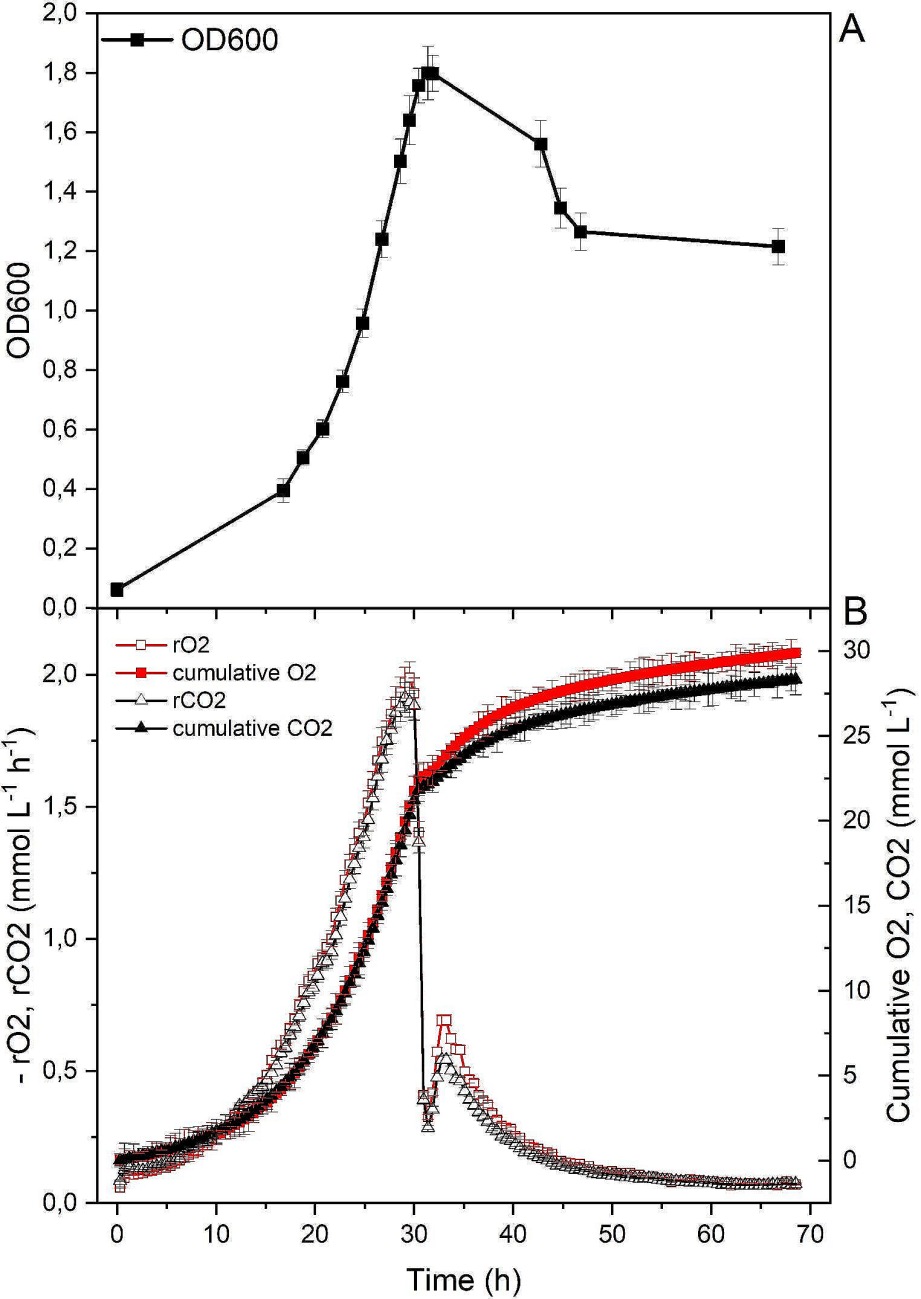
Gas analysis performed on *Acidiphilium* sp. SJH cultures during growth in medium with 10 mM (1.8 g/L) glucose. (**A**) Biomass concentration measured as optical density (OD_600_) and (**B**) O_2_ consumption (red line) and CO_2_ production (black line) rates and cumulative amounts of O_2_ and CO_2_

HPLC analysis showed that 2 g/L glucose, galactose and glycerol were completely depleted at the growth peak (Fig. 1A and C). Accordingly, gas analysis of the culture grown in glucose medium showed a close coupling of oxygen consumption and carbon dioxide production, as pinpointed by the measured RQ (respiratory quotient, i.e. the ratio of CO_2_ production to O_2_ consumption) value of 1, as expected for fully aerobic glucose metabolism. Moreover, the sharp drop in the respiratory activity, confirmed that substrate was completely exhausted (Fig. 2). Indeed, over the first 30 h of incubation, the growth of the cell culture (measured as optical density) and utilisation of the substrate (indicated as CO_2_ production and O_2_ consumption) were closely associated. The biomass yield was calculated from the linear correlation between OD and CO_2_ emission, and O_2_ profiles. The net amount of biomass, corresponding to 1.2 OD units, was produced by consuming 1 g/L of glucose. Approximately one half of the initial amount of glucose was used as energy source, therefore oxidised by bacterial catabolic reactions, as by CO_2_ emission, accompanied by equimolar (~ 30 mM) oxygen consumption. It can be deduced that the remaining part of the organic carbon supplied was incorporated into the biomass by assimilation reactions. The exponential growth phase with RQ = 1 was followed by a peak of O_2_ consumption/CO_2_ production with RQ value of 0.8 accompanied by biomass (OD_600_) decrease. This indicated the respiration of intracellular storage substance(s).

In cultures grown at higher glucose concentrations, consumption of the substrate was observed even after reaching the peak of growth. In the sample cultured in 5 g/L glucose medium, the carbon substrate was totally consumed 2 days after the growth peak. In the 10 g/L glucose culture, 3 g/L of the carbon source were still detected after 7 days of growth (Fig. 1D). HPLC measurements did not reveal the production of secondary metabolites or organic acids.

#### Bioleaching of iron impurities from quartz sand

**Fig. 3:**
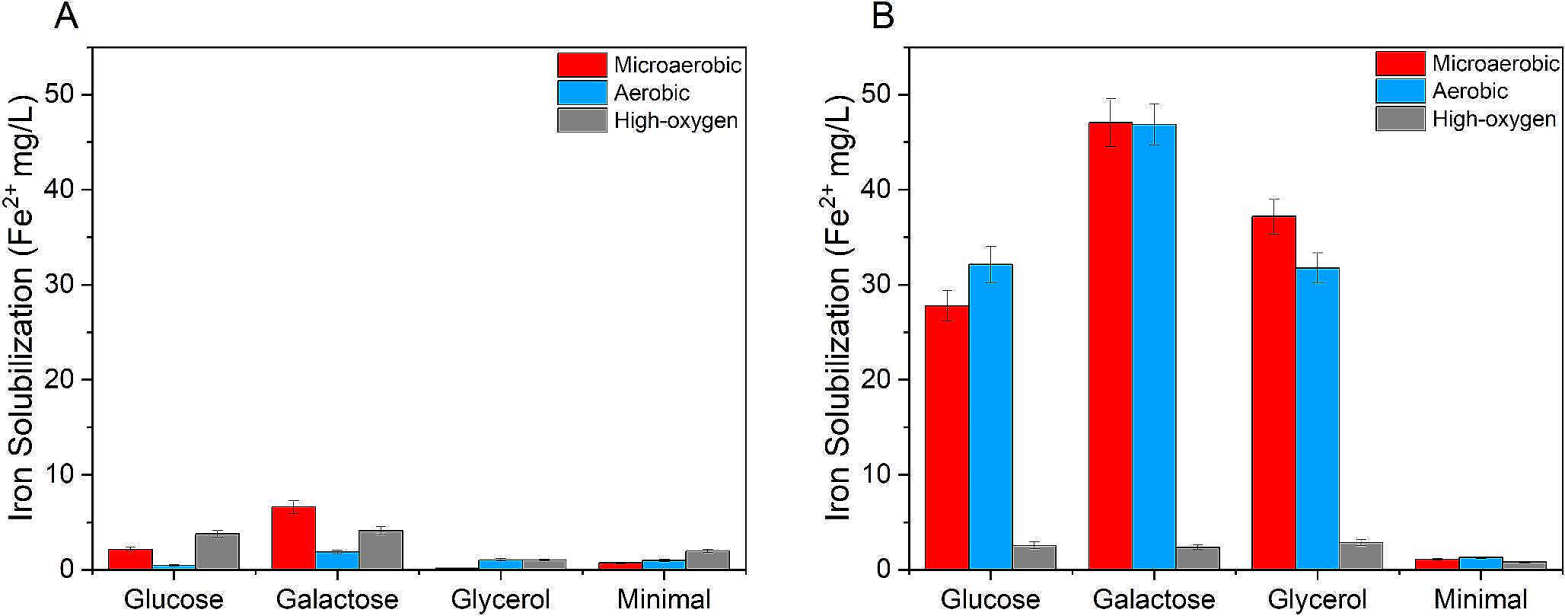
Bioleaching of iron impurities from quartz sand obtained with cells either in the stationary (**A**) or the exponential phase of growth (**B**) after four weeks of incubation with quartz sand. Measurements were performed in triplicate and reported as a mean ± standard deviation

The ability of *Acidiphilium* sp. SJH cells to reduce ferric iron was assessed by adding 40% quartz sand to cultures grown in different media up to either the stationary or the exponential phase of growth. Moreover, different aeration conditions were applied as described in the material and methods section (Fig. 3).

Stationary phase cells yielded poor iron solubilization and the best performing culture was the one cultivated in galactose medium. Highest iron recovery was 6.6 ± 0.7 mg/L, 1.9 ± 0.2 mg/L and 4.1 ± 0.4 mg/L after 4 weeks of incubation with quartz sand under microaerobic, aerobic and full aeration conditions respectively (Fig. 3A and Table 2). During bioleaching, cell growth was not detected (data not reported), since quartz sand was added to cells already in the stationary phase and no other nutrients were supplemented.

**Table 2:** Fraction of Fe^2+^ solubilised from quartz sand (w/w) under different conditions

Media	Stationary Phase Bioleaching (% Fe^2+^)			Exponential Phase Bioleaching (% Fe^2+^)		
	**Microaerobic**	**Aerobic**	**Full oxygenation**	**Microaerobic**	**Aerobic**	**Full oxygenation**
	%	%	%	%	%	%
Glucose	0.24 ± 0.02	0.05 ± 0.01	0.42 ± 0.04	3.10 ± 0.17	3.58 ± 0.21	0.28 ± 0.03
Galactose	0.74 ± 0.07	0.21 ± 0.01	0.46 ± 0.04	5.25 ± 0.28	5.23 ± 0.23	0.26 ± 0.02
Glycerol	0.02 ± 0.01	0.12 ± 0.01	0.11 ± 0.01	4.15 ± 0.20	3.54 ± 0.17	0.32 ± 0.03
Minimal	0.08 ± 0.01	0.11 ± 0.01	0.22 ± 0.02	0.12 ± 0.01	0.14 ± 0.01	0.09 ± 0.01

**Fig. 4:**
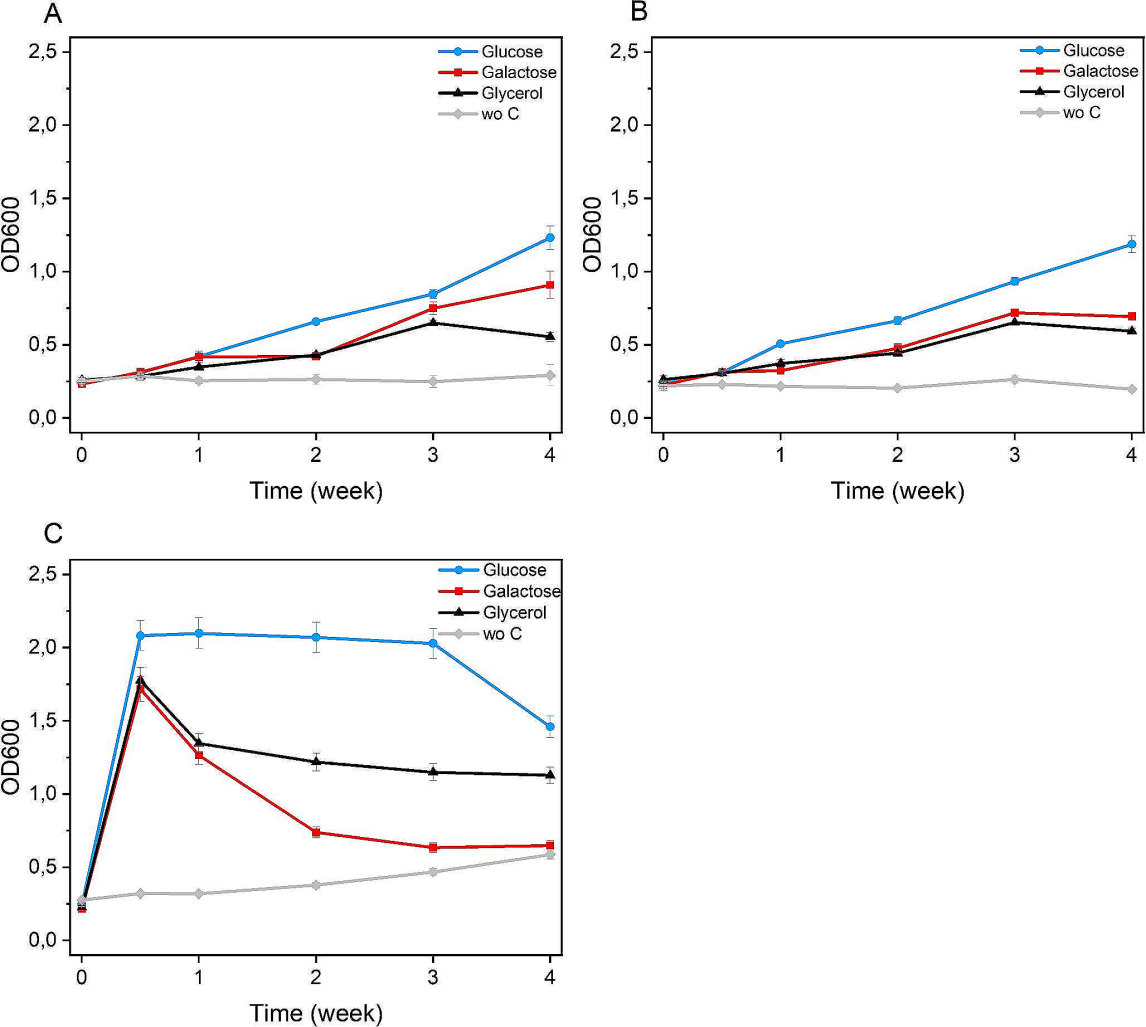
*Acidiphilium* sp. SJH growth during exponential phase bioleaching in microaerobic (**A**), aerobic (**B**) and full aeration (**C**) conditions. Measurements were performed in triplicate and reported as a mean ± standard deviation

In experiments performed with cells in the exponential phase of growth (Fig. 3B and Table 2), poor iron reduction was observed under full aeration conditions, whereas in microaerobic and aerobic conditions, cells grown in galactose media yielded highest iron recovery, respectively of 47 ± 2.4 mg/L and 46.8 ± 2.3 mg/L after 4 weeks of incubation with quartz sand (*p*-value < 0.01 in microaerobic and aerobic bioleaching condition between glucose, galactose, glycerol and minimal media, Tables S1 and S2). Fe^2+^ extraction occurred also in glucose and glycerol media, both in microaerobic and aerobic conditions, despite a lower yield. Doubling the biomass to sand ratio did not significantly increase iron solubilisation (data not shown). During exponential phase bioleaching, the cultures kept growing under all tested conditions (Fig. 4). Highest cell growth was detected under full aeration conditions (Fig. 4C), with 2.09 OD_600_ cell density achieved in glucose medium after 3 days of bioleaching. (*p*-value < 0.01 in full aerobic bioleaching condition between glucose, galactose, glycerol and minimal media, Table S3). In media supplemented with galactose or glycerol, the highest cell density was reached at the same time. Interestingly, the glucose-based culture maintained a constant density for over two weeks. In microaerobic and aerobic conditions, cell growth was reduced, although constant, over 4 weeks.

**Fig. 5:**
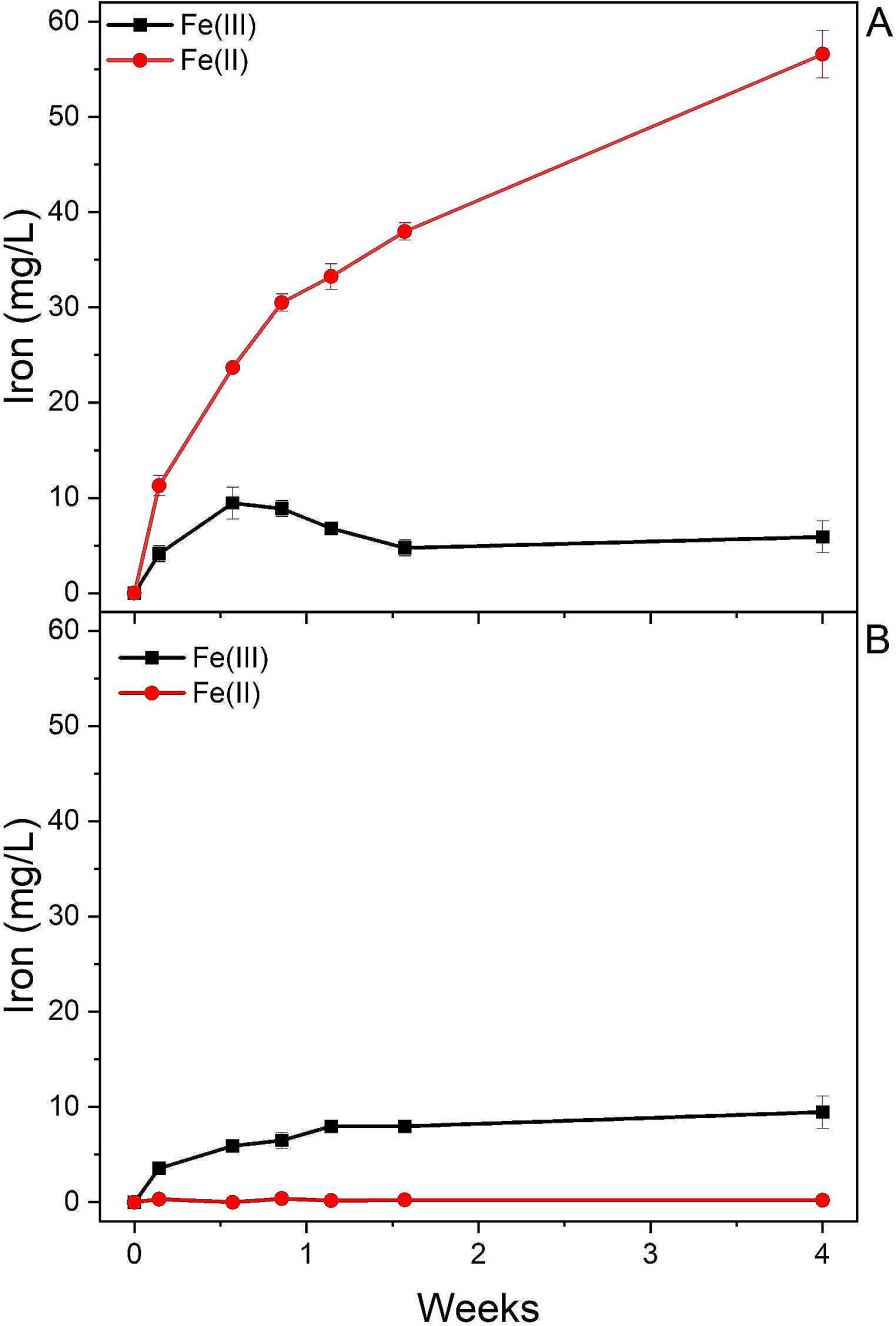
Extraction of iron from various iron-bearing minerals from the light fraction of the quartz sand under the same conditions used quartz sand with bacterial cells (**A**) and an abiotic control (**B**). Symbols indicate averages from triplicate flasks ± standard deviation

Since the light (heavy-minerals-free) fraction of quartz sand still contains iron impurities, in quantities that exceed the limits for the raw material for glass production (Table 1), in another series of experiments, the light product of gravity separation was subjected to bioleaching under the conditions previously defined as optimal (Fig. 5). In the presence of iron reducing bacteria most of the soluble iron was in divalent form. Total iron concentration in the leaching solutions reached approximately 55 mg/L after 4 weeks, which corresponded to a yield of 11% iron from the solid sample. The minute amounts of ferric iron in abiotic series likely resulted from dissolution of iron oxides in the raw material triggered by the acidic medium.

### Analysis of cell components by whole cells by Fourier-transform infrared (FTIR) spectroscopy

**Fig. 6:**
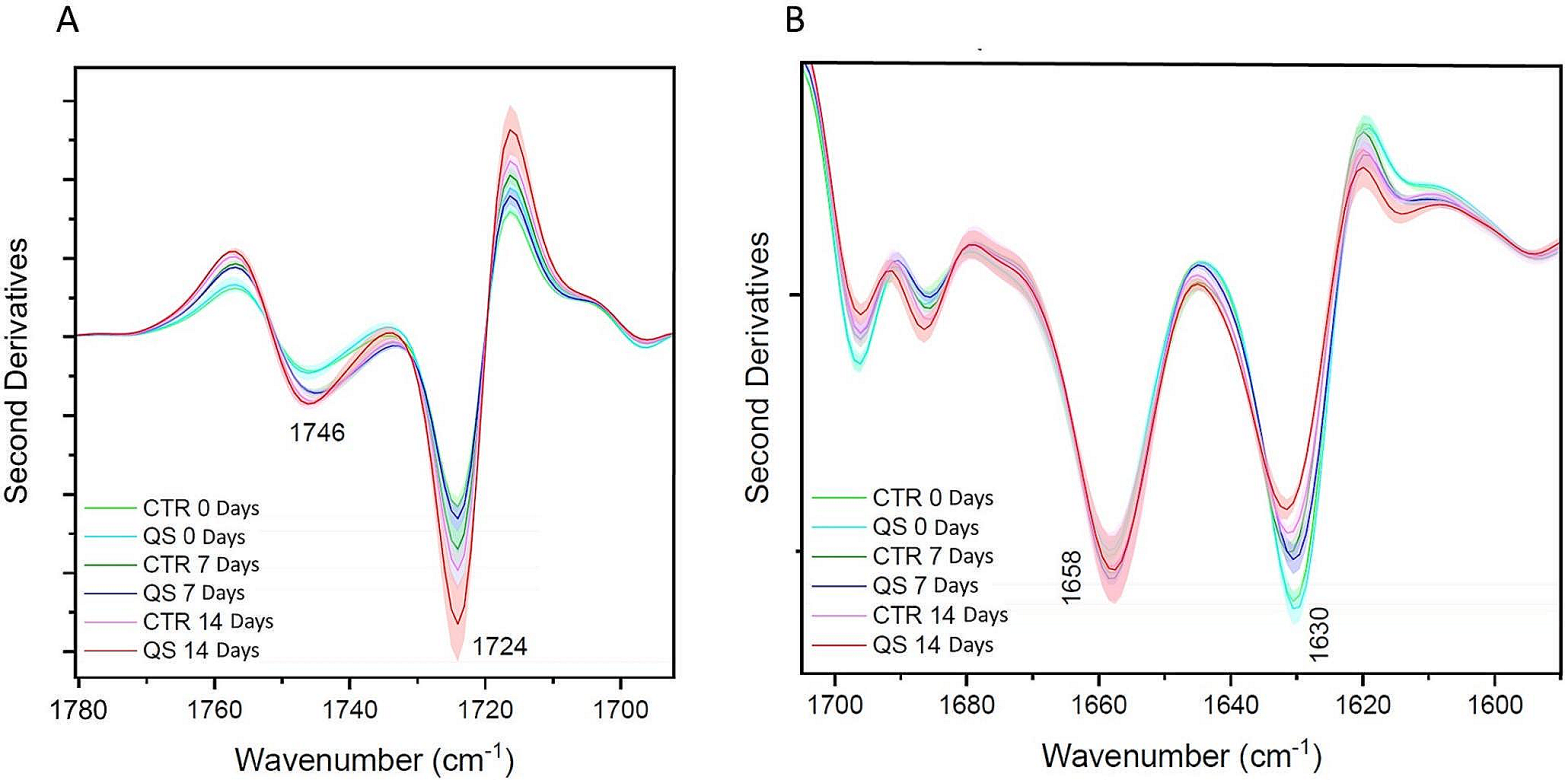
FTIR second derivative spectra of total lipid and total proteins of *Acidiphilium* sp. SJH whole cell. Total lipid signal at 1746 cm^-1^ wavenumber (carbonyl group, panel **A**) and total protein alpha-helix and beta-sheets signal, respectively at 1658 and 1630 cm^-1^ wavenumbers (**B**). CTR: Control condition, QS: Quartz Sand bioleaching condition

Iron bioleaching did not affect the lipid compositions of cells since the marker signal at 1746 cm^− 1^ wavenumber underwent similar changes in the presence and in the absence of quartz sand (Fig. 6A). Likewise, the signals responsive to total proteins (1658 and 1630 cm^− 1^ wavenumbers) were unaltered (Fig. 6B). It was also noticed that the signal corresponding to the alpha-helical component of the proteins (1658 cm^− 1^ wavenumber) did not change, while the one given by the beta component of the total proteins (1630 cm^− 1^ wavenumber) decreased. This behaviour, however, was similarly found in the control sample, indicating that the variation was not induced by the bioleaching conditions. On this basis, it was possible to conclude that the presence of quartz sand and the activity of cells in iron bioleaching do not significantly affect major cell macromolecular components.

**Fig. 7:**
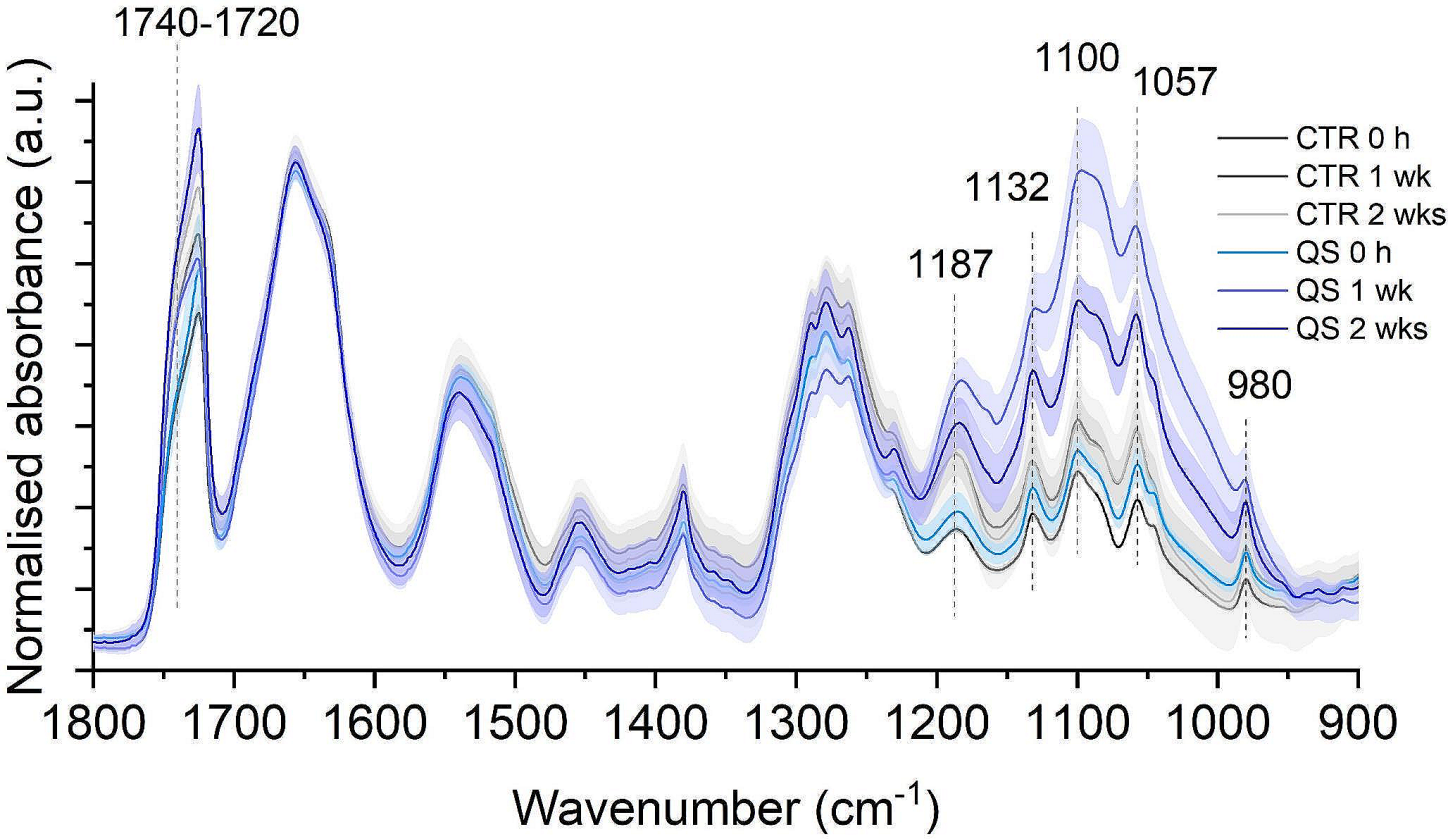
FTIR second derivative spectra monitored during bioleaching at different time points (time 0, after 1 week and after 2 weeks). 980, 1057, 1100, 1132, 1187 and 1740 − 1720 cm^-1^ are specific PHB wavenumbers. CTR: Control condition, QS: Quartz Sand bioleaching condition

**Fig. 8:**
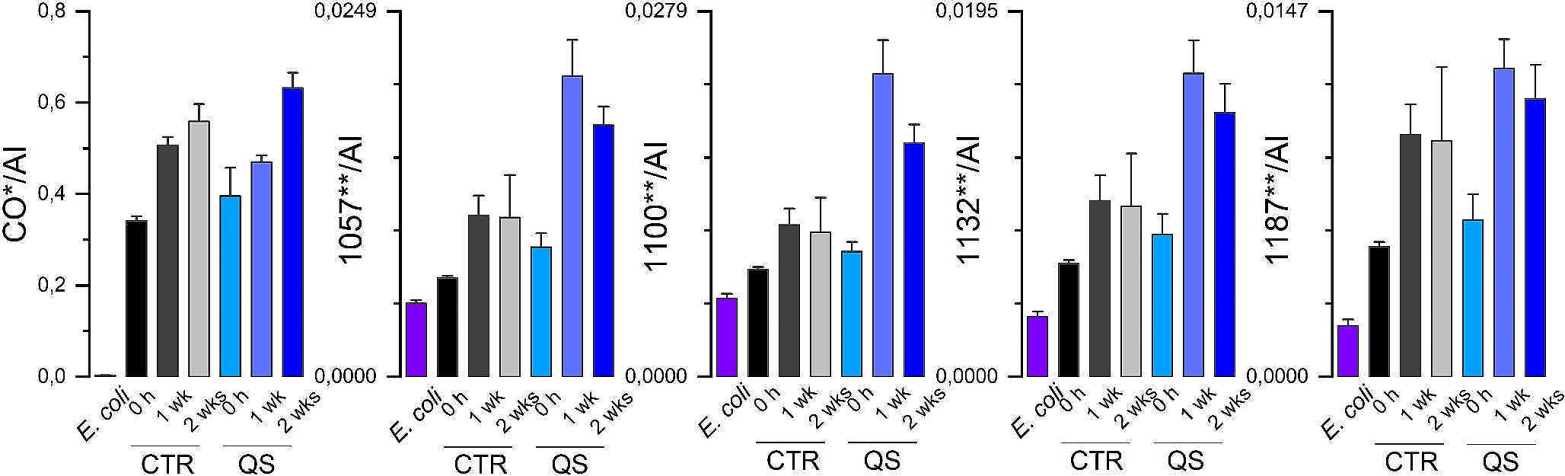
Intensity and variation over time of the peaks responsive to PHB recorded by FTIR. CTR: Control condition; QS: Quartz Sand bioleaching condition. *E. coli* signals were used as reference. *Peak Area; **Peak Intensity. CO: carbonylic group C = O (1740 − 1720 wavenumbers); AI: Amide I region (1700 − 1600 wavenumbers)

FTIR analysis highlighted the synchronised increase of peaks at specific wavenumbers, that is 980, 1057, 1100, 1132, 1187 and 1740 − 1720 cm^− 1^ (Figs. 7 and 8). The synchronous variation of these signals made it possible to assign them all to the same molecule, identified as poly-β-hydroxybutyrate (PHB) (Kansiz et al. 2000). FTIR analyses revealed the presence of PHB from the beginning of the bioleaching phase, suggesting that synthesis and accumulation of this polymer started during the biomass growth phase. Over time, the analyses monitored an increase of PHB after 7 and after 14 days of bioleaching. The synthesis and accumulation of PHB was verified not only under bioleaching conditions, but also in the cellular control in the absence of quartz sand, albeit at lower levels.

## Discussion

The aim of this work was to gain insight into the physiology of *Acidiphilium* sp. SJH during bioleaching of iron impurities. To this end different conditions of growth (carbon source, aeration condition) were tested and the relationships between biomass growth and bioleaching performance evaluated. This acidophilic bacterium was shown to grow on all provided carbon sources, albeit with different duplication rates. Glucose was the carbon source most rapidly depleted and supported fastest microbial growth, in comparison to galactose and glycerol. As expected, increasing glucose concentrations (5 g/L and 10 g/L) resulted in higher biomass growth. However, while in media containing up to 5 g/L glucose the carbon source was consumed by the day the highest cell density was reached, when growing at 10 g/L glucose cells consumed only 7 g/L, ruling out the hypothesis that a further increase could help achieve higher culture density before bioleaching. While it is difficult to comment on the behaviour of *Acidiphilium* sp. SJH on glucose, due to the paucity of data reported in the literature, the ability of this strain to uptake and consume galactose has been reported (Johnson and McGinness 1991b; Johnson 1995). Galactose is a carbon and energy source present in acidic environments, such as acid mines, mainly in the form of extracellular polymeric substances (EPS) (More et al. 2014; Saavedra et al. 2020). These compounds, mainly formed by polysaccharides, proteins, lipids, and extracellular DNA (Zafra et al. 2012; Li et al. 2020b) are synthesised by the various organisms that inhabit these environments such as *Ferroplasma acidiphilum* (Hossein Karimi Darvanjooghi et al. 2023), *Acidiphilium* sp. C61 (Li et al. 2020b) or *Acidithiobacillus ferrooxidans* (Barreto et al. 2005), as a defence against environmental stress. A galactose transporter (GalP) was identified in *Acidithiobacillus ferrooxidans*, where galactose is taken up by the microorganism but is not involved in metabolic pathways associated with energy and biomass production (Barreto et al. 2005). In contrast, in *Acidiphilium* sp. C61, genomic analyses showed the presence of transporters for ribose, fructose and xylose but not specific transporters for galactose. In this scenario, the genome analyses of *Acidiphilium* sp. SJH would be useful to unveil the metabolic pathways of this bacterium.

Although *Acidiphilium* sp. SJH is an aerobic organism, highest iron bioleaching yields have been obtained under microaerobic and aerobic conditions, while only small amounts of iron were solubilized in conditions of full aeration. Oxygen has been reported to play a key role in the reduction of ferric iron by *Acidiphilium* bacteria (Lonergan et al. 1996). However, different results have been described for different strains. *Acidiphilium rubrum* and *Acidiphilium acidophilum* have been shown to reduce Fe^3+^ at low oxygen levels (Hallberg and Barrie Johnson 2001; Johnson and Bridge 2002), whereas the same process has been reported to be independent on oxygenation in *Acidiphilium* sp. SJH, *Acidiphilium multivorum* and *Acidiphilium organovorum.* However, the results of this work showed that the reduction of ferric iron by *Acidiphilium* sp. SJH does depend on aeration, in fact it has been obtained under microaerobic and aerobic conditions, whereas it is poorly observed under full aeration. This could probably be explained by high oxygen competing with ferric iron as the final acceptor of the electron transport chain.

In this study, the dissolution of about 5% of the ferric ion present in the quartz sand has been achieved. The yield is lower than that reported in other works performed with the same bacterial strain but with different starting materials. For example Kupka et al. (2007), used the magnetic fraction of quartz sand, which contains about 25% ferric iron vs. 0.22% of the quartz sand used in this research. In another work, the use of ferric iron in soluble form achieved similar iron reduction yields but in only few hours of incubation (Johnson and Bridge 2002). At the best of our knowledge, this is the first report about the performances of *Acidiphilium* sp. SJH to in the bioleaching of iron impurities on unprocessed raw material.

The harsh and stressful conditions of acidic environments, in which *Acidiphilium* spp. live, led to molecular adaptations, including both physiological modifications at the protein and lipid levels (Tatsuzawa et al. 1996; Mirete et al. 2017) and the production of molecules and polymers with anti-oxidant, anti-stress or protective function (Rani et al. 2021; Müller-Santos et al. 2021; More et al. 2014).

FTIR spectroscopy was used to highlight possible stress-related changes in cellular components. This analysis did not reveal any major changes in total lipids and proteins composition triggered by pH or bioleaching. PHB production was instead assessed in all samples and increased in those performing bioleaching. Thus, while the presence of quartz sand in the cultures did not trigger a general stress response at the level of macromolecular components, the higher content of the protective PHB polymer during bioleaching would suggest that some specific stress was indeed evoked (Müller-Santos et al. 2021). More specifically a toxic effect of the released ferrous iron may be hypothesised with consequent oxidative stress (Bennett and Gralnik 2019; Müller-Santos et al. 2021).

Some other *Acidiphilium* species, such as *Acidiphilium sp.* C61 (Li et al. 2020b), *Acidiphilium* sp. DX1-1 (Xu et al. 2013), *Acidiphilium cryptum* JF-5 (Küsel et al. 1999; Xu et al. 2013) and *Acidiphilium* sp. JA12-A1 (Ullrich et al. 2015) have been reported as capable of producing PHB. PHB producing bacteria can be divided into two groups (Lee 1996): the first one accumulates PHB during the stationary growth phase when nutrients are unbalanced, whereas the second group produces PHB during the growth regardless of nutrients ratio (Xu et al. 2013).

This is the first report of PHB production and accumulation by *Acidiphilium* sp. SJH, that can be included in the second group of PHB-producing bacteria. This feature, together with its ability to use various carbon sources, might support the introduction of *Acidiphilium* sp. SJH in a circular economy concept, where sugar-rich waste biomasses, such as molasses, might be the starting point of a parallel bioleaching & PHB production process.

### Electronic supplementary material

Below is the link to the electronic supplementary material.

Supplementary Material 1

## Data Availability

No datasets were generated or analysed during the current study.
