# Supplementary material for: Iron bioleaching and polymers accumulation by an extreme acidophilic bacterium
Source: Arch Microbiol. 2024 May 22;206(6):275. doi: 10.1007/s00203-024-04005-4 (PMC11111502; doi:10.1007/s00203-024-04005-4)
Supplement: Supplementary file 1 — Supplementary Material 1 [file 203_2024_4005_MOESM1_ESM.pdf]

Supplementary Material

p-value results from the statistical analyses performed as a two-tail t-Test of Fe^{2+} release between different carbon sources in different aeration conditions (table S1 and table S2) and of growth between different carbon sources in different aeration conditions (table S3).

Supplementary Table S1.

p-value from t-Test analyses of Fe^{2+} release in exponential phase bioleaching after 4 weeks of incubation

**Medium Comparation**	***p*-value of Exponential Phase Bioleaching**		
	**Microaerobic**	**Aerobic**	**Full Aerobic**
Glucose-Galactose	0,0003	0,0005	0,6317
Glucose-Glycerol	0,0039	0,6955	0,1946
Glucose-Minimal	0,0006	0,0007	0,0003
Galactose-Glucose	0,0003	0,0005	0,6317
Galactose-Glycerol	0,0031	0,0004	0,1057
Galactose-Minimal	0,0005	0,0005	0,0001
Glycerol-Glucose	0,0039	0,6955	0,1946
Glycerol-Galactose	0,0031	0,0004	0,1057
Glycerol-Minimal	0,0009	0,0007	0,0005

Supplementary Table S2.

p-value from t-Test analyses of Fe^{2+} release in stationary phase bioleaching after 4 weeks of incubation

**Media Comparation**	**p-value of Stationary Phase Bioleaching**		
	**Microaerobic**	**Aerobic**	**Full Aerobic**
Glucose-Galactose	0,0036	0,0027	0,0387
Glucose-Glycerol	0,0023	0,0004	0,0004
Glucose-Minimal	0,0019	0,0130	0,0001
Galactose-Glucose	0,0036	0,0027	0,0387
Galactose-Glycerol	0,0031	0,0061	0,0002
Galactose-Minimal	0,0032	0,0032	0,0001
Glycerol-Glucose	0,0023	0,0004	0,0004
Glycerol-Galactose	0,0031	0,0061	0,0002
Glycerol-Minimal	0,0043	0,7746	0,0083

Supplementary Table S3.

p-value from t-Test analyses of growth during exponential phase bioleaching at grow peak

**Media Comparation**	**p-value of Growth in Exponential Phase Bioleaching**		
	**Microaerobic**	**Aerobic**	**Full Aerobic**
Glucose-Galactose	0,0048	0,0002	0,0043
Glucose-Glycerol	0,0003	0,0001	0,0067
Glucose-Minimal	0,0002	0,0000	0,0004
Galactose-Glucose	0,0048	0,0002	0,0043
Galactose-Glycerol	0,0067	0,0477	0,4479
Galactose-Minimal	0,0020	0,0004	0,0003
Glycerol-Glucose	0,0003	0,0001	0,0067
Glycerol-Galactose	0,0067	0,0477	0,4479
Glycerol-Minimal	0,0007	0,0002	0,0002
